# Research on Chromium-Free Passivation and Corrosion Performance of Pure Copper

**DOI:** 10.3390/ma18194585

**Published:** 2025-10-02

**Authors:** Xinghan Yu, Ziye Xue, Haibo Chen, Wei Li, Hang Li, Jing Hu, Jianli Zhang, Qiang Chen, Guangya Hou, Yiping Tang

**Affiliations:** 1 College of Material Science and Engineering, Zhejiang University of Technology, Hangzhou 310014, China; yxh20020703@163.com (X.Y.); lhzjut@163.com (H.L.); hujing05022023@163.com (J.H.); zhangjianli711@163.com (J.Z.); cq415@zjut.edu.cn (Q.C.); houguangya@zjut.edu.cn (G.H.); 2 Zhejiang Gpilot Technology Co., Ltd., Wenzhou 325699, China; xueziye@cnjiabo.com; 3 Shandong Hesheng Copper Co., Ltd., Dongying 257029, China; liw@londianwason.com

**Keywords:** pure copper bonding wire, chromium-free passivation, environment protection, corrosion resistance

## Abstract

In response to the actual needs of pure copper bonding wires, it is crucial to develop a chromium-free passivator that is environmentally friendly and has excellent corrosion resistance. In this study, three different composite organic formulations of chromium-free passivation solutions are selected: 2-Amino-5-mercapto-1,3,4 thiadiazole (AMT) + 1-phenyl-5-mercapto tetrazolium (PMTA), 2-mercaptobenzimidazole (MBI) + PMTA, and Hexadecanethiol (CHS) + sodium dodecyl sulfate (SDS). The performance analysis and corrosion mechanism were compared with traditional hexavalent chromium passivation through characterization techniques such as XRD, SEM, and XPS. The results show that the best corrosion resistance formula is the combination of the PMTA and MBI passivation agent, and all its performances are superior to those of hexavalent chromium. The samples treated with this passivation agent corrode within 18 s in the nitric acid drop test, which is better than the 16 s for Cr^6+^ passivation. The samples do not change color after being immersed in salt water for 48 h. Electrochemical tests and high-temperature oxidation test also indicate better corrosion resistance than Cr^6+^ passivation. Through the analysis of functional groups and bonding, the excellent passivation effect is demonstrated to be achieved by the synergistic action of the chemical adsorption film formation of PMTA and the anchoring effect of MBI. Eventually, a dense Cu-PMTA-BMI film is formed on the surface, which effectively blocks the erosion of the corrosive medium and significantly improves the corrosion resistance.

## 1. Introduction

Owing to its exceptional electrical and thermal conductivity, cost-effectiveness, and high mechanical strength, pure-copper bonding wire has been extensively adopted as an internal interconnect in integrated circuits, diodes, and transistors, progressively supplanting conventional gold wire to satisfy the demands of high-density packaging [1,2]. Nevertheless, the intrinsically elevated chemical reactivity of copper renders it susceptible to pronounced corrosion and discoloration in electrolytes containing Cl^−^, SO_4_^2−^, or NO_3_^−^ ions and at high temperatures. Such degradation not only diminishes the electrical conductivity and compromises the mechanical integrity of the bonding wire but also engenders potential safety hazards that can jeopardize the operational stability of electronic devices [3,4]. In order to effectively mitigate the corrosion of pure-copper bonding wires and enhance their stability under complex service environments, extensive research efforts have been directed toward various surface modification strategies for copper. These include spraying, surface solid-solution deposition, electroplating, chemothermal treatment, and chemical passivation [5,6,7,8,9], all of which aim to form a protective film on the Cu surface to isolate the substrate from the external environment, thereby achieving corrosion protection. Among these, chemical passivation—particularly the traditional chromate immersion process—has been predominantly employed. In this method, a passivation film is formed on the copper surface via immersion in chromic acid solutions, effectively shielding the metal from atmospheric oxygen and moisture and preventing undesirable chemical reactions. However, hexavalent chromium (Cr^6+^) is a well-documented carcinogen and exhibits significant environmental toxicity, leading to stringent regulatory restrictions on its industrial use worldwide [10]. Consequently, the development of an environmentally benign, chromium-free passivation technology has become an urgent imperative in the field of copper surface engineering.

In recent years, chromium-free passivation strategies for metallic substrates have bifurcated into two principal paradigms: inorganic passivation and organic passivation [11,12,13]. Monocomponent passivating agents generally yield films that are non-uniform and microporous; accordingly, synergistic combinations of two or more passivators are routinely exploited to attain cooperative enhancement of protective efficacy. Regarding inorganic passivation, Wu et al. [14] investigated the effect of molybdate on spontaneous passivation of low-carbon steel and a novel 10% Cr steel in saturated Ca(OH)_2_ solution. They found that molybdate promoted spontaneous passivation of low-carbon steel in the early stages of exposure, and its passivation ability could be further enhanced with increasing molybdate concentration. Xu et al. [15] conducted a comparative study on brass substrates employing sodium tungstate (Na_2_WO_4_) in conjunction with polyaspartic acid; optimization at a polyaspartic acid/Na_2_WO_4_ mass ratio of 1:1 yielded superior corrosion inhibition relative to either component used in isolation. Nevertheless, the elevated cost of certain inorganic salts—molybdates in particular—constitutes a significant economic barrier to large-scale industrial deployment.

Some research progress has also been made in the field of organic passivation, wherein chelating functionalities within organic molecules facilitate the formation of hydrophobic, metal-coordinated protective films. Huang et al. [16] proposed the synthesis of a water-soluble and highly efficient corrosion inhibitor PBTB containing a monobenzotriazole moiety and a copper based PDBTB containing a dibenzotriazole moiety in a 3.5 wt % sodium chloride solution. The measurement results showed that PDBTB exhibited a greater corrosion inhibition effect than PBTB. Yang et al. [17] employed a pomelo-peel extract to generate a protective layer on copper through concurrent physisorption and chemisorption and delineated the attendant corrosion inhibition mechanism. Mo et al. [18] synthesized Al^3+^-doped BTA-Cu composite films on electrolytic copper foil via a primary passivation system composed of NaAlO_2_ and BTA. Despite these achievements, the majority of reported formulations entail intricate compositions and/or multistage post-treatments, thereby impeding facile translation to industrial practice.

Through the analysis of the interactions between the functional groups of commonly used organic passivators and different metal matrix forms, as well as the required concentrations during use, three excellent passivation formulas were obtained after conducting permutations and orthogonal experiments on several commonly used passivators. This study investigated the combined passivation process of these three organic passivators (AMT + PMTA, MBI + PMTA and CHS + SDS) [19,20,21]. Through a facile chemical-immersion process, dense, chromate-free passivation films were formed on Cu substrates. Comparative assessment of corrosion resistance and oxidation stability against conventional Cr^6+^-based passivation was performed. Comprehensive characterization encompassing microstructural morphology, surface phase composition, and interfacial chemical bonding was carried out. The resultant passivation bath is characterized by a minimalist formulation and low toxicity, thereby providing pivotal guidance and practical utility for large-scale industrial implementation.

## 2. Materials and Methods

### 2.1. Raw Materials and Passivation Formulations

The starting material was 8-mm-diameter bonding-grade copper wire (≥99.99 wt % Cu). Cylindrical specimens (Ø 8 mm × 5 mm) were precision-sectioned from this stock using a DMW3020-BB multifunctional water-jet cutter (Jiangsu Nanjing. Nanjing Dadi Waterjet Co., Ltd.). The observed object in the experiment was the cross-sectional area of the sample. The more copper crystal planes exposed made the reaction more intense and the contrast effect more obvious.

Table 1 summarizes the four passivation baths employed in this study. Baths 1, 2 and 3 are chromium-free formulations, whereas Bath 4 is the conventional chromic-acid reference. For conciseness, copper wires treated with Baths 1, 2, 3 and 4 will hereafter be designated Samples A, B, C and D, respectively. The process of preparing the solution is as follows:

**A**: Dissolve 0.15 g of AMT in a small amount of ethanol, directly add 0.5 g of PMTA to 1 L of deionized water, then add the AMT ethanol solution to the PMTA aqueous solution while constantly stirring. Ultrasonic treatment for 10 min to ensure the solution is uniform.

**B**: Dissolve 0.25 g of MBI in a small amount of ethanol, directly add 1 g of PMTA to 1 L of deionized water, then add the MBI ethanol solution to the PMTA aqueous solution while constantly stirring. Ultrasonic treatment for 10 min to ensure the solution is uniform.

**C**: Add 10 g of CHS to 1 L of deionized water, then add 7.5 g of SDS to the same solution. Stir continuously to ensure the solution is uniform.

**D**: Add 5 g of CrO_3_ to 1 L of deionized water, then add 0.5 g of H_2_SO_4_ to the same solution. Stir for 30 min to ensure the solution is uniform.

### 2.2. Pre-Treatment and Passivation Methods

The specimens were subjected to a combined surface pretreatment and chemical immersion passivation protocol. First, acid pickling was performed by immersing the as-received samples in a 10 wt % H_2_SO_4_ solution for 30 s to remove the native oxide layer. Subsequently, the samples were ultrasonically cleaned in de-ionized water for 5 min to eliminate residual acid and surface contaminants. After cleaning, the specimens were immersed for 30 s in the respective passivation baths to form a protective film. Following passivation, the samples were again ultrasonically rinsed in de-ionized water for 5 min to remove any residual passivation solution. Finally, the rinsed specimens were dried in a vacuum oven at 25 °C for 1 h.

### 2.3. Testing and Characterization Instruments

Macroscopic surface morphology was examined using a high-resolution CCD microscope (Model GP-660V, Jiangsu Suzhou Kunshan Gaopin Precision Instrument Co., Ltd.). Phase evolution after high-temperature oxidation tests was identified by X-ray diffraction (RIGAKU D/Max 2550 PC, Japan). Microstructural features were imaged with a field-emission scanning electron microscope (FESEM, Hitachi S-4700, Japan) operated at 15 kV. The chemical state of the principal elements within the passivation film and the valence change in the underlying Cu were determined by X-ray photoelectron spectroscopy (XPS, Axis Ultra DLD, Shimadzu, Japan); additionally, the O 1s spectral profile was employed to evaluate the protective efficacy of the film.

### 2.4. Testing Methods

#### 2.4.1. Nitric Acid Drop Test

The nitric acid drop test is a widely accepted method for assessing the corrosion resistance of passivated surfaces. By monitoring the latency, intensity and morphology of bubble formation following acid exposure, the protective efficacy of the film can be rapidly evaluated. In this study, a 10 μL droplet of HNO_3_:H_2_O (1:2, *v*/*v*) was dispensed onto the cross-sectional surface of each specimen. A high-definition CCD microscope was employed to observe the droplet, and the elapsed time from initial contact to the onset of visible bubbling was recorded as the failure criterion.

#### 2.4.2. Electrochemical Measurements

Electrochemical measurements were carried out on a Shanghai Chenhua CHI760D workstation in a three-electrode cell employing a platinum foil counter electrode, a saturated calomel reference electrode, and the passivated bonding-copper wire as the working electrode with an exposed area of 0.9 cm^2^; after 20 min of immersion in naturally aerated 3.5 wt % NaCl, the open-circuit potential was monitored for 300 s, electrochemical impedance spectra were recorded at the stabilized OCP with a 5 mV amplitude over 10 kHz–0.01 Hz, and Tafel polarization curves were swept from −250 mV to +250 mV versus OCP at 10 mV·s^−1^; corrosion potential (E_corr_) and corrosion current density (i_corr_) were obtained by extrapolating the linear portions of the anodic and cathodic Tafel branches to η = 0 (E = E_corr_), the intersection on the log i axis giving log i_corr_ and hence i_corr_ [22].

#### 2.4.3. Salt-Spray Immersion Weight-Loss Test

A 3.5 wt % NaCl solution was employed to simulate high-salinity conditions. In a saltwater environment, the corrosion process on the surface of copper is usually relatively rapid in the early stage. The 24 h time point can capture the changes in the initial stage of corrosion, while the 48 h time point can reflect the stability and durability of the passivation film over a longer period. By comparing the data of 24 h and 48 h, the performance changes in the passivation film in a continuous corrosive environment and its long-term protective effect on the copper substrate can be evaluated. Surface corrosion of each specimen was visually monitored after 24 h and 48 h of immersion, and weight loss was measured to quantify the extent of passivation-film damage and the corresponding corrosion rate [23].

#### 2.4.4. High-Temperature Oxidation Resistance Test

To simulate the high-temperature conditions encountered during storage, transportation and service of bonding copper wires, an oxidation-resistance test was designed with a stepped thermal profile. Samples subjected to the four passivation treatments (A, B, C and D) were individually exposed to 100 °C, 120 °C, 140 °C and 180 °C for 1 h in a muffle furnace. After each temperature step, the coupons were air-cooled to 25 °C, and their oxidation behavior was compared by (i) macroscopic colourimetry and CCD imaging, (ii) micro-morphological inspection via FESEM, and (iii) phase identification using XRD, thereby providing a direct assessment of the relative antioxidative performance of each passivation system under progressively severe thermal stress [24].

## 3. Results and Discussion

### 3.1. Corrosion Resistance at Room Temperature

**Figure 1:**
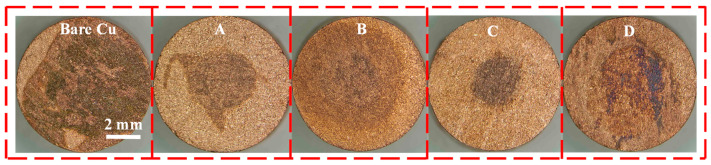
The macroscopic morphology of the sample (Bare copper, sample A treated with AMT and PMTA, sample B treated with MBI and PMTA, sample C treated with CHS and SDS, and sample D treated with chromic acid) after Nitric Acid Drop Test.

Figure 1 presents the macroscopic surface morphologies after nitric acid droplet exposure, and Table 2 lists the incubation periods required for bubble initiation on the cross-sectional surface of the bonding copper wires. Evidently, the extent of corrosion varies among the specimens. The bare copper, Sample A, and Sample D exhibit large and deeply corroded areas; in particular, the bare copper shows pronounced radial expansion of the attacked zone. Conversely, although Samples B and C also sustain corrosion, both the severity and the affected area are markedly smaller. Consistently, Table 2 indicates that Sample B begins to evolve bubbles only after 18 s—outperforming the 16 s recorded for Sample D—demonstrating superior resistance to nitric acid penetration and corroborating the visual evidence in Figure 1.

**Figure 2:**
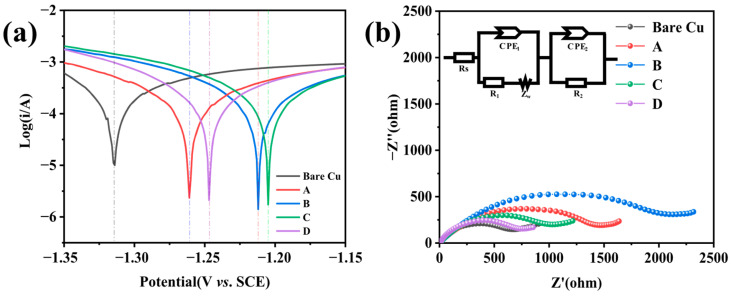
(**a**) Tafel polarization curves and (**b**) EIS Nyquist plots of the passivated specimens.

The study investigated the effect of different passivation formulations on the corrosion resistance of samples in a 3.5 wt % NaCl solution, with the corresponding Tafel polarization curves shown in Figure 2a. When using Tafel curves to evaluate the corrosion resistance of metals, the corrosion potential (E_corr_) and corrosion current density (i_corr_) are crucial parameters. The corrosion potential is mainly influenced by the thermodynamic properties of the material itself and its surface condition. A higher E_corr_ value indicates a lower tendency for the material to corrode, while a lower E_corr_ value suggests a higher corrosion tendency. However, E_corr_ only indicates the likelihood of corrosion [25]. The corrosion current density (i_corr_) is closely related to the corrosion rate, with a larger icorr indicating a faster corrosion reaction rate. Once a corrosion reaction occurs, the extent of corrosion is only related to the corrosion rate, so i_corr_ is the primary parameter to consider [26]. E_corr_ is only considered when the differences in i_corr_ are not significant. Figure 2a shows that the corrosion current of samples after chromium-free passivation and chromate passivation is significantly lower than that of bare copper, indicating that both passivation processes provide good protection and enhance the corrosion resistance of the samples in corrosive media. According to the data in Table 3, the differences in E_corr_ among the passivation films are not significant. However, when comparing i_corr_, sample B has the lowest corrosion current density, at 2.442 × 10^−4^ A·cm^−2^,which is better than sample D’s 3.543 × 10^−4^ A·cm^−2^. This indicates that the passivation layer on sample B has a higher resistance, effectively reducing i_corr_ and improving the corrosion resistance of sample B. Owing to the formation of the surface passivation film, the i_corr_ of Samples A and C was reduced, thereby decelerating the corrosion rate of the samples. Simultaneously, the i_corr_ values of Samples A and C were 3.719 × 10^−4^ A·cm^−2^ and 3.444 × 10^−4^ A·cm^−2^ respectively. These values were slightly lower than that of Sample D, indicating that both Samples A and C also exhibited relatively outstanding performance.

Considering that the same substrate has the same internal resistance, different passivation films have similar double-layer characteristics, and all tests are conducted in a 3.5 wt % NaCl solution system, the surface charge transfer resistance of different samples can be obtained through EIS testing. EIS tests were conducted on all specimens and the spectra were fitted to the equivalent circuit shown in Figure 2b; the corresponding Nyquist plots consist of a single capacitive arc followed by an upward-sloping Warburg line. During fitting, two constant-phase elements (CPE) were introduced to account for non-ideal capacitive behavior at two distinct interfaces. R_s_ denotes the electrolyte resistance; R_1_, Z_w_ and CPE_1_ describe the charge-transfer and diffusion processes occurring at the passive film/electrolyte interface, governed by both concentration and activation polarization. R_2_ and CPE_2_ characterize the charge-transfer process between the passive film and the copper substrate [27]. The radius of the capacitive arc in the high-frequency region reflects interfacial kinetics: a larger arc implies a higher charge-transfer resistance (R_ct_) and thus slower charge migration [28]. Sample B exhibits the largest arc radius, signifying a marked increase in R_ct_; Table 4 confirms that Sample B possesses the highest R_ct_ of 2096 Ω, substantially exceeding the 749 Ω of Sample D. Consequently, the passive film on Sample B delivers the best corrosion protection. At the same time, sample A with an R_ct_ of 1464 Ω and sample C with an R_ct_ of 1027 Ω can also confirm the results obtained from the Tafel curve, and can correspond to the above salt water immersion test results. Both show a lower interface ion transport rate than sample D.

**Figure 3:**
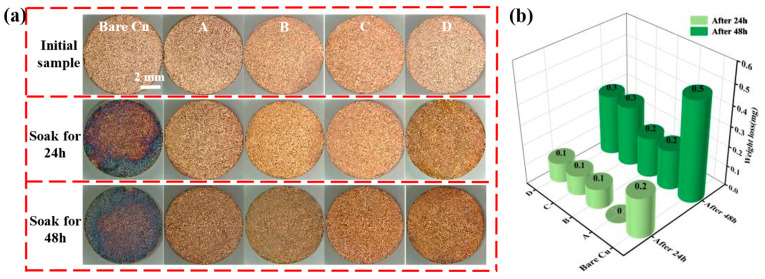
(**a**) Macroscopic corrosion morphology of samples (Bare copper, sample A treated with AMT and PMTA, sample B treated with MBI and PMTA, sample C treated with CHS and SDS, and sample D treated with chromic acid) after saltwater immersion and (**b**) weight loss after immersion.

Figure 3a shows the macroscopic appearance of samples subjected to different passivation treatments after immersion in 3.5 wt % NaCl solution for 24 h and 48 h. After 24 h of immersion, the bare copper sample exhibited severe edge corrosion with unevenly distributed corrosion products. Samples A and D showed slight discoloration, but to a lesser extent than the bare copper. In contrast, no visible changes were observed for Samples B and C. After 48 h of immersion, the bare copper surface was entirely covered with corrosion products. Samples A and D developed minor pitting, while Samples B and C remained largely unchanged, demonstrating superior corrosion resistance. Figure 3b illustrates the weight loss after immersion. After 24 h, the bare copper sample lost 0.2 mg, whereas Samples B, C, and D each lost 0.1 mg. No weight loss was detected for Sample A due to the precision limit of the measuring instrument. After 48 h, Samples A and B exhibited a weight loss of 0.2 mg, while Samples C and D lost 0.3 mg, and the bare copper sample lost 0.5 mg. These results indicate that Sample B’s passivation film maintained higher integrity after corrosion, effectively protecting the copper substrate.

The above data indicate that the passivation film on the surface of sample B has excellent acid and salt corrosion resistance at room temperature, reflecting that the No. 2 passivator meets the passivation standard of copper wire at room temperature.

### 3.2. Oxidation Resistance at High Temperatures

**Figure 4:**
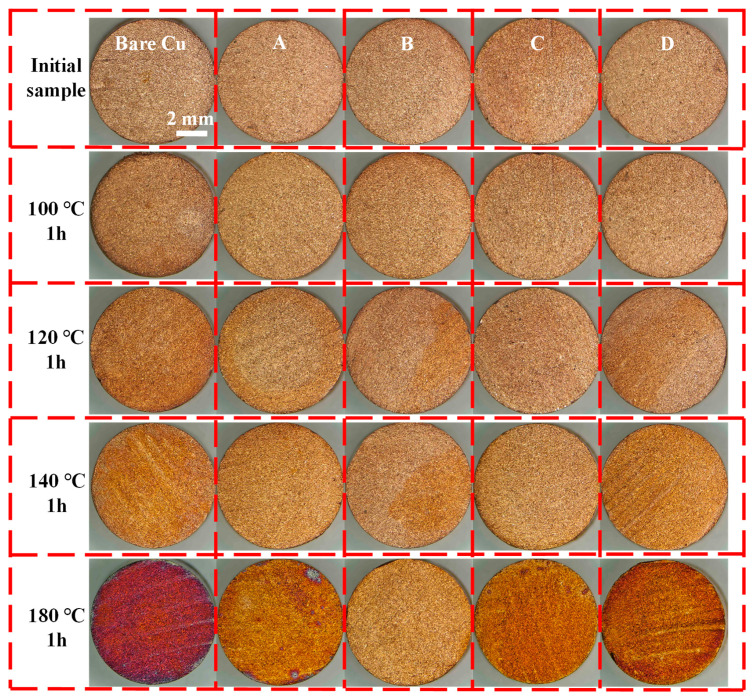
Macroscopic surface morphology of samples (Bare copper, sample A treated with AMT and PMTA, sample B treated with MBI and PMTA, sample C treated with CHS and SDS, and sample D treated with chromic acid) after exposure to different temperatures for 1 h.

The high-temperature oxidation resistance of bonding copper wires after treatment with different passivation baths was investigated by heating the samples in a tube furnace for 1 h at temperature steps of 100 °C, 120 °C, 140 °C, and 180 °C. The macroscopic surface morphology, with a focus on oxidation discoloration, is shown in Figure 4. As the temperature increases, both the bare copper and passivated samples exhibit varying degrees of oxidation. The bare copper sample shows significant darkening of the entire cross-section at 120 °C, whereas passivated samples display only edge oxidation, partial discoloration, or minor pitting. At 140 °C, the bare copper sample further darkens, and Samples A, C, and D become fully oxidized, while Sample B shows only partial discoloration. At 180 °C, the bare copper cross-section loses its luster entirely, turning deep purplish-red with pronounced oxide formation. Among the passivated samples, Sample D shows the most severe discoloration, whereas the color of samples A and C has deepened again, and there are severely oxidized parts that can be observed with the naked eye. Sample B, however, undergoes the least color change compared to the initial state. This indicates that, in the absence of a protective passivation layer, the substrate oxidizes more rapidly at elevated temperatures. Among the passivation schemes tested, Sample B demonstrates the best high-temperature oxidation resistance.

**Figure 5:**
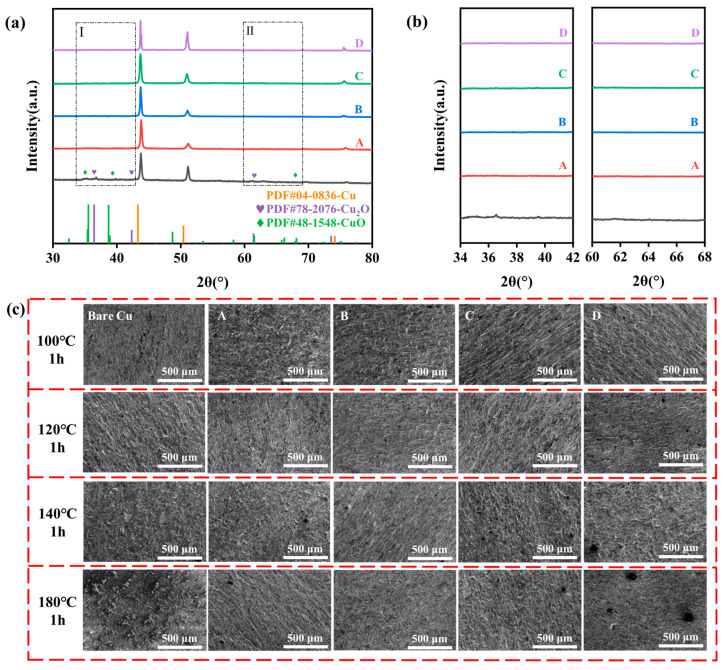
(**a**) XRD patterns of samples (Bare copper, sample A treated with AMT and PMTA, sample B treated with MBI and PMTA, sample C treated with CHS and SDS, and sample D treated with chromic acid) after treatment at 180 °C for 1 h, (**b**) enlarged view of selected regions of the spectra, and (**c**) SEM images of sample surfaces after exposure to different temperature steps.

Figure 5a presents the XRD patterns of the samples after 1 h of exposure to 180 °C. All five samples display diffraction peaks at 2θ values of approximately 43.29°, 50.43°, and 74.13°, corresponding to the (111), (100), and (110) crystal planes of Cu (Cu, PDF#04-0836). Except for the bare copper sample, no other distinct peaks are observed on the surfaces of the passivated samples. The enlarged XRD spectra in Figure 5b reveal that the bare copper sample exhibits characteristic diffraction peaks of Cu_2_O at 36.44° and 61.41° (Cu_2_O, PDF#78-2076), and CuO at 32.50° and 61.52° (CuO, PDF#48-1548). These peaks are absent in the other samples, indicating that the passivation treatments effectively prevent significant phase transformation on the sample surfaces under high-temperature conditions. The thin passivation layer may also attenuate the intensity of the diffraction peaks by masking the underlying oxide layers. Figure 5c shows the SEM images of the sample surfaces after high-temperature oxidation resistance tests under different conditions. The surfaces, initially disordered and uneven due to water-jet cutting, exhibit varying degrees of oxidation. The bare copper sample accumulates a dense layer of oxide particles at 180 °C after 1 h, whereas Samples A, C, and D show fewer oxide particles. Notably, Sample B displays almost no oxide particles on its surface. This observation suggests that the passivation film formed by Sample B effectively blocks the ingress of oxidizing species, thereby providing superior oxidation resistance.

According to the test results, the excellent high-temperature oxidation resistance demonstrated by sample B proves that Passivator No. 2 meets the conditions for copper wire use at high temperatures.

### 3.3. Component Analysis

**Figure 6:**
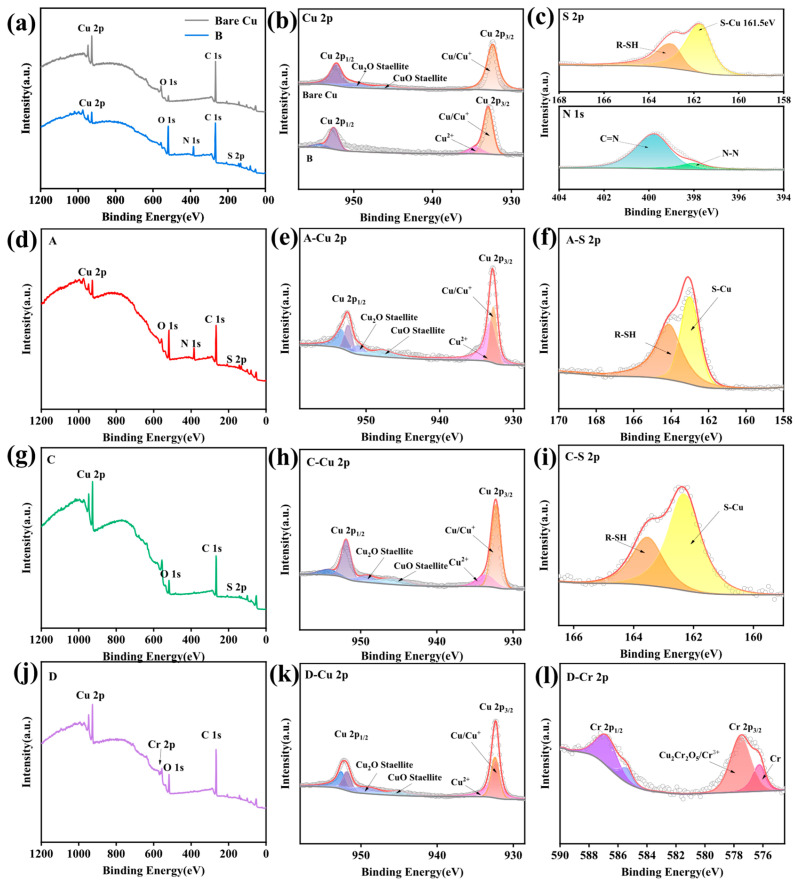
XPS spectra: (**a**) survey scan of bare Cu, (**b**) Cu 2p of bare Cu; (**c**) N 1s and S 2p of Sample B; survey scans of (**d**) Sample A, (**g**) Sample C, (**j**) Sample D; Cu 2p of (**e**) Sample A, (**h**) Sample C, (**k**) Sample D; S 2p of (**f**) Sample A and (**i**) Sample C; and Cr 2p of (**l**) Sample D.(The circular lines represent the fitted curves, and the red lines represent the original curves).

XPS was employed to probe the evolution of surface elemental chemistry before and after passivation. Figure 6 presents the complete data set: survey scans and Cu 2p regions for bare Cu and Sample B, high-resolution N 1s and S 2p regions for Sample B, and comparative survey, Cu 2p, S 2p and Cr 2p spectra for Samples A, C and D. Survey scans (Figure 6a) reveal that Sample B contains Cu, O, S, N and C, whereas bare Cu shows only Cu, O and C, confirming the presence of a polymer film derived from PMTA and MBI. Cu 2p high-resolution spectra (Figure 6b) display for bare Cu a single component at 932.6 eV corresponding to Cu^0^/Cu^+^ (ΔBE ≈ 0.1 eV) [29]. Sample B shows an additional peak at 934.8 eV attributable to Cu^2+^, yet the absence of the characteristic CuO satellite band (940–945 eV) indicates that the divalent copper is coordinated to organic ligands rather than present as CuO [30]. S 2p and N 1s spectra of Sample B (Figure 6c) corroborate this interpretation. The S 2p_3/2_ component at 161.5 eV evidences S–Cu bonding, whereas the 163.4 eV peak corresponds to residual thiol (R-SH) [31]. In the N 1s region, peaks at 397.9 eV (N=N) and 399.8 eV (C-N) confirm the integrity of the azole moieties within the polymer film. Equivalent analyses for Samples A, C and D are provided in Figure 6d–l. Survey scans (Figure 6d,g,j) outline the elemental composition of each film, while Cu 2p spectra (Figure 6e,h,k) display the same Cu^0^/Cu^+^/Cu^2+^ signatures. S 2p spectra for Samples A and C (Figure 6f,i) exhibit analogous S-Cu and R-SH doublets to those of Sample B. Figure 6l presents the Cr 2p spectrum of Sample D prepared by the conventional chromate passivation process; the component at 577.4 eV corresponds to Cr^3+^, whose trivalent chromium compounds confer high mechanical strength and thus act as the structural backbone of the passive film, and at 576.3 eV, it corresponds to the fitting peak of Cr [32], because CrO_3_ reacts with copper to generate CuO and Cr:

CrO_3_ + 3Cu = 3CuO + Cr, (1)

4CrO_3_ + 4CuO = 2Cu_2_Cr_2_O_5_ + 3O_2_ (2)

**Figure 7:**
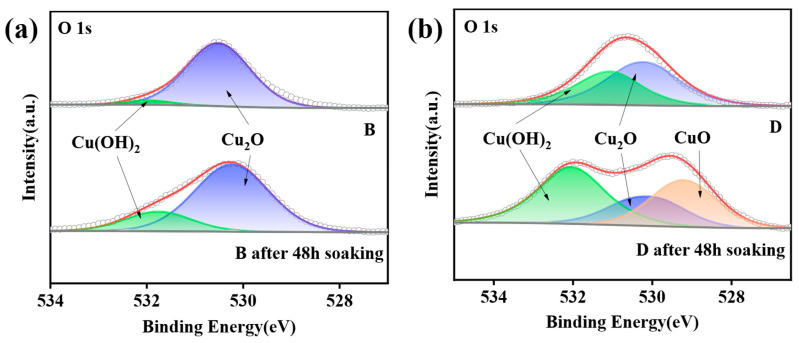
O 1s spectra of (**a**) Sample B and (**b**) Sample D before and after 48 h of saltwater immersion.

To compare the passivation efficacy with the conventional CrO_3_ process, the surface oxygen chemical states of Samples B and D were analyzed before and after 48 h of saltwater immersion. Figure 7 presents the O 1s spectra of the two samples before and after immersion. In Figure 7a, Sample B exhibits two peaks at 530.43 eV (Cu_2_O) and 531.89 eV (O in -OH) prior to immersion. Considering possible trace formation of Cu(OH)_2_ during passivation via reaction of Cu with water, no new phases emerge after 48 h of immersion; only the -OH peak area slightly increases, indicating no Cu oxidation. In contrast, Sample D (Figure 7b) shows peaks at 530.47 eV (Cu_2_O) and 531.15 eV (Cu(OH)_2_) before immersion. Post-immersion, the O 1s spectrum splits, with a new, large peak at 532.41 eV corresponding to CuO [29], indicating substantial CuO formation on the surface, consistent with Figure 3. These results confirm that the Cu-PMTA-MBI passivation film effectively blocks water and oxygen ingress, and Sample B’s chromium-free passivation outperforms the traditional chromate process.

Through the above XPS analysis, the elemental state and bonding condition on the sample surface can be understood. At the same time, it is confirmed that the No. 2 passivator achieved a relatively excellent passivation effect during the salt water immersion experiment. (The circular lines represent the fitted curves, and the red lines represent the original curves)

### 3.4. Passivation Mechanism Analysis

**Figure 8:**
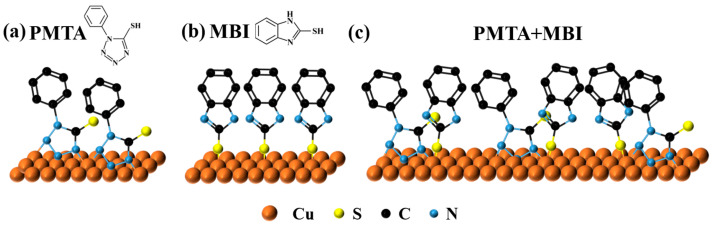
(**a**) Structural formula of PMTA and its bonding model with the substrate; (**b**) structural formula of MBI and its bonding model with the substrate; (**c**) synergistic effects of PMTA and MBI.

Based on the comprehensive performance analysis, Sample B achieved the best passivation effect. Its passivation bath consists of 1-phenyl-5-mercapto-tetrazole (PMTA) and 2-mercaptobenzimidazole (MBI). The azazole ring and thiol bond carried in the components deserve attention. According to the conclusion drawn from XPS, the bond interaction between them and the matrix is the key to the formation of the passivation film. The formation of the passivation film is primarily determined by coordination between molecules and surface adsorption. The lone pairs of electrons on the N atoms in the azole ring structure can coordinate with the d-orbital vacancies in the metal atoms. The azole structure readily bonds with Cu to form a dense, three-dimensional passivation film covering the copper surface, providing a protective passivation effect [33,34,35]. PMTA molecules, with their tetrazole structure, also coordinate their N lone pairs with the empty d orbitals of Cu. This chemisorption covers the bonding copper wire surface, forming a Cu-PMTA passivation film (Figure 8a). Additionally, thiol groups can form strong coordination bonds with metals after oxidation and dehydrogenation, creating monothiolates and one-dimensional polymer films adsorbed on the copper surface [36,37]. Similarly, MBI, which also has a thiol group, can undergo the same bonding reaction with metallic copper, generating Cu-MBI monothiolates that anchor the passivation film firmly to the copper surface like rivets (Figure 8b). During passivation, the two-dimensional network formed by PMTA and Cu, combined with the anchoring effect of MBI, work synergistically to form a Cu-PMTA-MBI passivation film (Figure 8c). These bonds connect the metal and organic molecules, firmly attaching them to the copper surface. They not only block external oxygen and moisture but also alter the chemical properties of the metal surface, reducing reactive sites that are prone to oxidation. This minimizes the formation of oxidation products and prevents the metal from reacting with corrosive substances in the environment, thereby maintaining its performance and ensuring long-term stability.

## 4. Conclusions

This study found that the sample treated with Passivation Bath No. 2, composed of MBI and PMTA, exhibited the best corrosion resistance. The sample only began to corrode after 18 s of high-concentration nitric acid drop-test, and electrochemical testing and saltwater immersion experiments confirmed that it has corrosion resistance comparable to that of Cr^6+^ passivation. After high-temperature oxidation resistance testing (180 °C, 1 h), the sample still met the requirement of almost no discoloration. Through the analysis of the component mechanism of the passivation solution, it is concluded that the Cu-PMTA-BMI passivation film formed by the chemical adsorption film of PMTA and Cu and the anchoring synergy of the strong coordination bond between MBI and Cu determines the excellent passivation effect. Compared with Cr^6+^-passivated samples, the content of CuO and Cu(OH)_2_ on the surface of the sample before and after saltwater immersion was almost zero, showing excellent passivation effect. The components of the passivation agent are readily available, low-cost, environmentally friendly, and renewable, making it a potential candidate for the corrosion protection of metallic copper, in line with the requirements of green chemistry and environmental engineering.

## Figures and Tables

**Table 1:** Passivation solution number and composition.

Number	Solution Composition
**1**	0.15 g/L AMT + 0.5 g/L PMTA
**2**	0.25 g/L MBI + 1 g/L PMTA
**3**	10 g/L CHS + 7.5 g/L SDS
**4**	5 g/L CrO_3_ + 0.5 g/L H_2_SO_4_

**Table 2:** The nitric acid drip results.

Sample	Bare Cu	A	B	C	D
**Drip results (s)**	8	16	18	14	16

**Table 3:** Tafel test results.

Sample	Bare Cu	A	B	C	D
**E_corr_ (V vs. SCE)**	−1.314	−1.212	−1.261	−1.205	−1.247
**i_corr_ (A** **·** **cm^−2^)**	8.917 × 10^−4^	3.719 × 10^−4^	2.442 × 10^−4^	3.444 × 10^−4^	3.543 × 10^−4^

**Table 4:** Charge-transfer resistances (R_ct_) for the different specimens.

Sample	Bare Cu	A	B	C	D
**R_ct_ (** **Ω** **)**	654	1464	2096	1027	749

## Data Availability

The original contributions presented in this study are included in the article. Further inquiries can be directed to the corresponding author.

## Abbreviations

The following abbreviations are used in this manuscript:

AMT	2-Amino-5-mercapto-1,3,4-thiadiazole
PMTA	1-phenyl-5-mercapto tetrazolium
MBI	2-mercaptobenzimidazole
CHS	Hexadecanethiol
SDS	Sodium dodecyl sulfate
